# Prostate Cancer Presenting With an Unusual Presentation of Rectal Pain and Bleeding

**DOI:** 10.7759/cureus.18766

**Published:** 2021-10-14

**Authors:** Muhammad S Haq, Pravin M Thomas, Marcos Almonte, Vinuta Mohan

**Affiliations:** 1 Internal Medicine, Saint Francis Medical Center, Trenton, USA; 2 Internal Medicine: Endocrinology, Saint Francis Medical Center, Trenton, USA

**Keywords:** metastastic cancer, rectal mass, rectal bleeding, colorectal cancer, prostate cancer

## Abstract

We report an atypical case of prostate cancer with rectal involvement presenting with gastrointestinal symptoms predominately and a rectal mass. A 51-year-old male patient came to the hospital with abdominal pain and rectal bleeding. Imaging revealed prostate enlargement, perirectal lymphadenopathy, and multiple hepatic and pulmonary nodules. The patient also had an elevated prostate-specific antigen (PSA) of 502 ng/mL (against normal range 0.6-0.7 ng/mL). Biopsies were performed on tissue samples taken from the rectum and prostate gland, which confirmed the diagnosis of prostate adenocarcinoma. The lack of urinary symptoms and close clinical similarity to colorectal cancer presented a diagnostic challenge for us. Familiarity with this specific presentation of prostate cancer is necessary to avoid misdiagnoses and guide correct treatment.

## Introduction

Prostate cancer is a devastatingly common malignancy in men, second only to skin cancer, and its incidence is increasing day by day [1]. Although common, prostate cancer rarely presents with rectal involvement, despite its proximity to the rectum. We present a rare case of prostate cancer presenting with gastrointestinal symptoms and a rectal mass mimicking colorectal cancer. Considering prostate cancer early in the differential diagnosis of such a presentation can decrease diagnostic delays and guide treatment.

## Case presentation

A 51-year-old caucasian male with a past medical history of astigmatism and prediabetes was referred to our hospital from the state prison with the chief complaint of lower abdominal pain. According to the patient, the pain had started almost six months ago, it was 9.5/10 in intensity, crampy in nature, and associated with per rectal bleeding and dark brown color stools mixed with blood. Aside from the abdominal pain, the patient had also experienced constipation, intermittent nausea, and vomiting and had noticed a weight loss of about 35 lbs in the last six months. He also recently started experiencing lower back pain that would radiate bilaterally down the thighs and to the legs. The patient did not endorse a family history of any malignancy. Vitals on admission included a blood pressure of 166/97, respiratory rate of 18 breaths per minute on room air, a pulse of 71 bpm (beats per minute), and a temperature of 36.8 degrees Celsius. Physical exam was significant for tenderness in the hypogastric region without rebound, guarding, and positive bowel sounds. Based on the clinical presentation alone, the patient was worked up for pathologies involving the gastrointestinal tract. 

**Figure 1:**
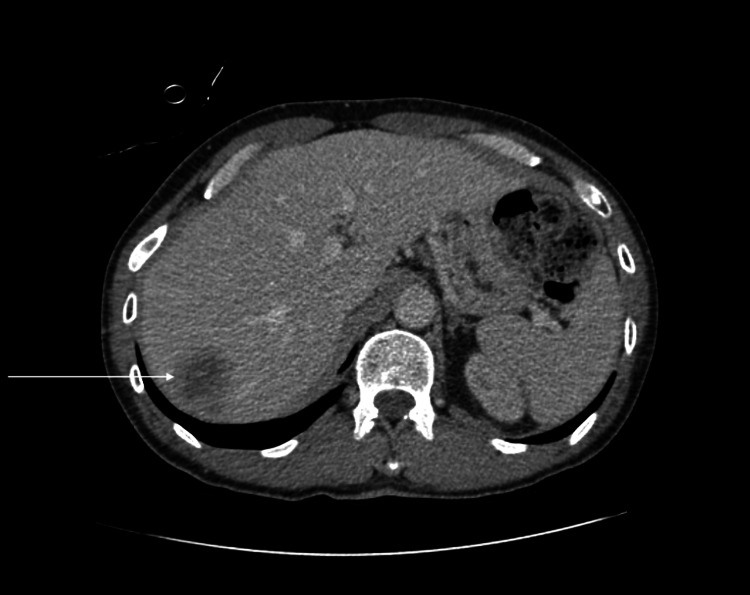
CT scan of the abdomen showing indeterminate complex hypodense lesion at the posterior right hepatic lobe measuring 3.6 x 3.6 cm (white arrow)

**Figure 2:**
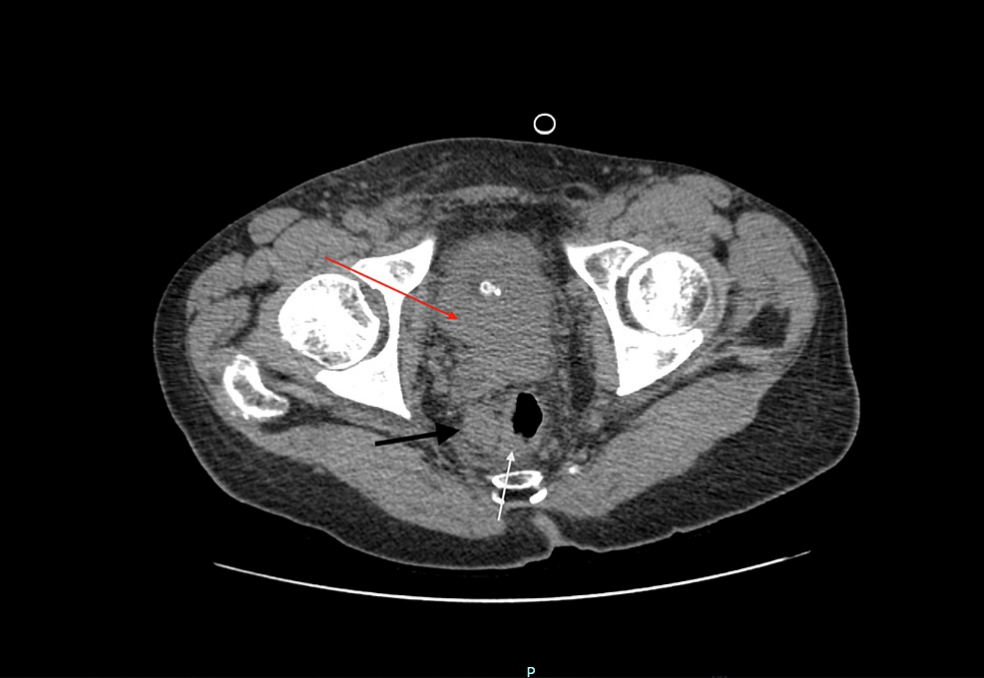
CT scan of the abdomen White arrow shows enlarged and irregular prostate; Black arrow shows prominent perirectal lymph node; Red arrow shows bladder wall thickening

His initial laboratory investigation results were significant for creatinine of 1.75 mg/dL (normal range 0.7-1.3 mg/dL) with estimated glomerular filtration rate (eGFR) of 41 mL/min/1.73m^2^, alkaline phosphatase of 469 U/L (normal range 34-104 U/L), iron of 26 ug/dL (normal range 50-212 ug/dL) with ferritin of 386 ng/mL (normal range 24-336 ng/mL), total iron-binding capacity (TIBC) of 357 (normal range 250-450), and hemoglobin of 10.8 g/dL (normal range 12.9-16.7 g/dL). His PSA (prostate-specific antigen) was found to be high at 502 ng/mL (normal range 0.6-0.7 ng/mL) with a CEA (carcinoembryonic antigen) of 1.4 ng/mL (normal range 0-2.5 ng/mL) and cancer antigen (CA)-125 of 27 U/mL (<46 is normal). A CT scan of the abdomen done on admission revealed an indeterminate complex hypodense lesion at the posterior right hepatic lobe measuring 3.6 x 3.6 cm (Figure 1), bilateral hydronephrosis and hydroureter with an enlarged and irregular prostate, and multiple prominent perirectal lymph nodes measuring up to 2.1 x 1.6 cm within the right perirectal area (Figure 2).

**Figure 3:**
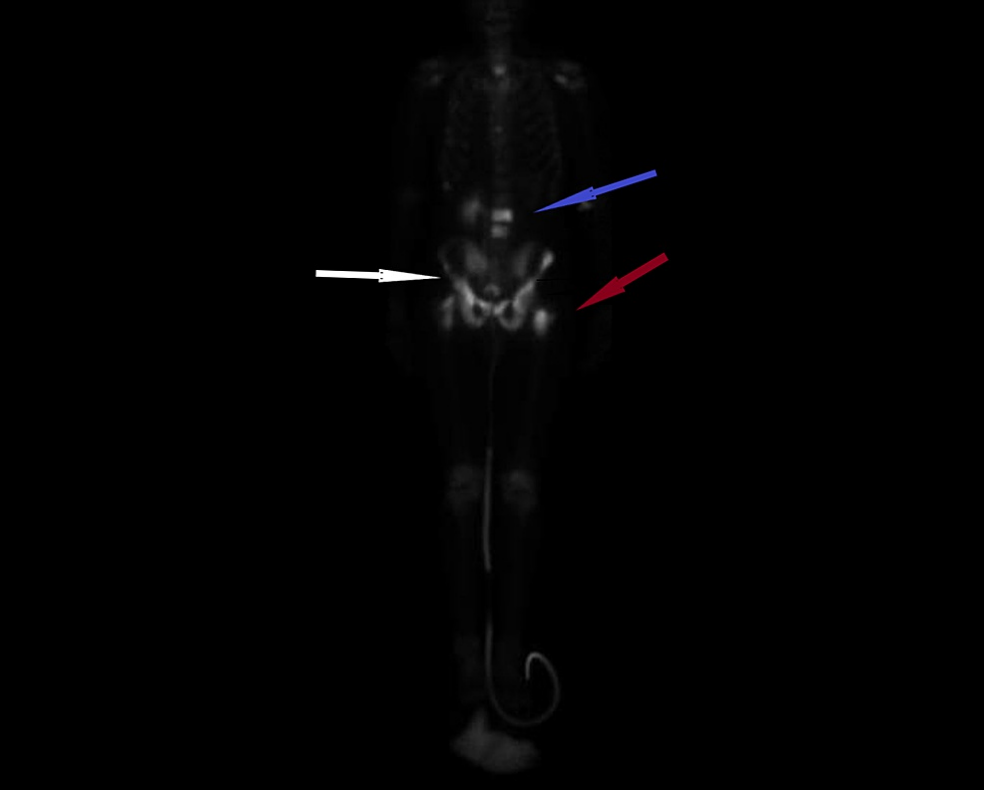
Bone scan showing increased uptake in lumbar (L1, L2) and thoracic (T11, T12) vertebrae (Blue arrow), right sacrum (White arrow), and bilateral proximal femurs (Red arrow)

CT scan of the chest showed a 4 mm nodule within the medial right lower lobe, scattered small nodules along the right major fissure, and ground glass and nodular airspace opacity in the left lower lobe. MRI of the pelvis revealed an infiltrative mass arising from the prostate gland measuring 4 x 5 cm. It also showed the tumor invading the posterior bladder trigone, seminal vesicles, and the ventral wall of the anal rectal junction with extension to the lower rectum. The diagnosis of metastatic cancer was confirmed, and a subsequent workup was done to find out the extent of metastasis. The bone scan revealed multiple metastatic lesions involving lumbar (L1, L2) and thoracic (T11, T12) vertebrae, right sacrum, and bilateral proximal femurs (Figure 3).

After having confirmed metastatic cancer involving multiple organ systems, the next task was to figure out the primary origin of the tumor. For that reason, a colonoscopy and prostate biopsy was done as we believed primary cancer would be of either colonic or prostatic origin. Colonoscopy showed a large hard mass in the rectum. Biopsies were taken and sent for histopathology, which revealed metastatic prostatic adenocarcinoma. Histochemical staining of the sample revealed PSA and prostatic acid phosphatase (PSAP) positivity, and the Gleason score was calculated to be at least an 8. A subsequent prostate biopsy later confirmed this diagnosis and revealed a Gleason score of 9, with grade group 5. 

As our patient presented with advanced prostate cancer with distant metastasis, the initial treatment of choice for which is hormonal therapy, he was started on leuprorelin, a gonadotrophin-releasing hormone analog, and bicalutamide, an androgen receptor blocker. The patient is currently being followed in the hematology/oncology clinic for further management. He is to return in three weeks’ time for the second dose of leuprorelin. Further management will be based on the patient’s response to the treatment. 

## Discussion

Prostate cancer is a leading cause of cancer-associated deaths in men around the world and metastatic disease has the worst prognosis [1,2]. Prostate cancer can metastasize to several locations, including regional lymph nodes, liver, and bones, but there are only a few published reports of it metastasizing to the gastrointestinal tract (outside the liver) [3,4]. The exact mechanism of metastasis to the gastrointestinal tract is unknown but it most likely involves seeding through the bloodstream or the lymphatics. In our patient, prostate cancer had metastasized to the lungs, liver, rectum, and bones by the time he presented to our hospital, making finding the source of origin a challenge.

Prostate cancer in most patients presents as an incidental finding on screening PSA tests and digital rectal examinations, and some men might go through their whole life without ever knowing they had prostate cancer, only to be diagnosed on post-mortem autopsies [5-7]. When symptoms do arise, they usually involve the lower urinary tract. Some of the more common symptoms include lower urinary tract obstruction, hematuria, and erectile dysfunction [5,6]. Another way prostate cancer can present is through paraneoplastic syndromes (PNS), and in some cases, it might be the first manifestation of the disease [8]. Our patient, surprisingly, did not have any of these typical symptoms on presentation. Instead, he presented with symptoms that initially made us believe that we were dealing with gastrointestinal malignancy. It was only after histopathological studies that we were confidently able to establish the diagnosis of prostate adenocarcinoma.

Prostate cancer with rectal involvement can present a diagnostic conundrum, delaying diagnosis and management. Distinguishing the etiology of a rectal mass, specifically distinguishing prostate cancer from colorectal cancer, is necessary to guide correct treatment. Prostate cancer with rectal involvement is rare and has a poor prognosis according to the literature [8]. Common symptoms of prostate cancer with rectal involvement, according to one literature review, include: constipation, abdominal pain, rectal bleeding, and diarrhea [9]. These symptoms are also often seen as “alarm symptoms” concerning colorectal cancer [10]. 

Not surprisingly, case reports have confirmed a tendency and risk of misdiagnosis in such presentations of prostate cancer. One paper documented nine cases of prostate cancer involving the rectal wall being misdiagnosed as rectal cancer [11]. Of those nine cases, only one of the patients had prostate-specific symptoms, such as changes in urination [11]. There are other cases of near misses of prostate cancer with rectal involvement occurring in presentations of abdominal pain and hematochezia wherein ischemic colitis co-existed with rectally involving prostate cancer [12]. Furthermore, diagnoses of co-synchronous cases of colorectal cancer and prostate cancer have been reported, further complicating the picture [13].

## Conclusions

As discussed above, prostate cancer can present in a variety of different ways. Unlike some other malignancies, screening for prostate cancer has yielded conflicting results and is not currently widely used. It is therefore essential to meticulously examine and investigate all cases where prostate involvement is suspected. Prostate cancer with rectal involvement may mimic colorectal cancer. A predominance of gastrointestinal symptoms, a rectal mass, and a lack of urinary symptoms may lead to significant diagnostic delays. Even if the presentation is atypical, prostate cancer should be considered in the differential diagnoses of cases of rectal masses.

## References

[1] Tian JY, Guo FJ, Zheng GY, Ahmad A (2018). Prostate cancer: updates on current strategies for screening, diagnosis and clinical implications of treatment modalities. Carcinogenesis.

[2] Wang G, Zhao D, Spring DJ, DePinho RA (2018). Genetics and biology of prostate cancer. Genes Dev.

[3] Gandaglia G, Abdollah F, Schiffmann J (2014). Distribution of metastatic sites in patients with prostate cancer: a population-based analysis. Prostate.

[4] Mahjoubi Z, Bibi M, Hedhli H, Ouanes Y, Rhouma SB, Yassine N (2020). A case report of prostate cancer presenting as a symptomatic pelvic mass mimicking lymphoma. Int J Surg Case Rep.

[5] Merriel SW, Funston G, Hamilton W (2018). Prostate cancer in primary care. Adv Ther.

[6] Hong MK, Kong J, Namdarian B (2010). Paraneoplastic syndromes in prostate cancer. Nat Rev Urol.

[7] Miller DC, Hafez KS, Stewart A, Montie JE, Wei JT (2003). Prostate carcinoma presentation, diagnosis, and staging: an update form the National Cancer Data Base. Cancer.

[8] Llarena Ibarguren R, Zabala Egurrola JA, Arruza Echevarría A (1993). Rectal involvement secondary to prostatic adenocarcinoma (Article in Spanish). Arch Esp Urol.

[9] Bowrey DJ, Otter MI, Billings PJ (2003). Rectal infiltration by prostatic adenocarcinoma: report on six patients and review of the literature. Ann R Coll Surg Engl.

[10] Rasmussen S, Haastrup PF, Balasubramaniam K (2019). Predictive values of colorectal cancer alarm symptoms in the general population: a nationwide cohort study. Br J Cancer.

[11] Tang T, Yang Z, Zhang D, Qu J, Liu G, Zhang S (2017). Clinicopathological study of 9 cases of prostate cancer involving the rectal wall. Diagn Pathol.

[12] Obana T, Kishimoto M (2021). A case of circumferential rectal wall thickening caused by prostate cancer invasion concomitant with ischemic colitis (Article in Japanese). Nihon Shokakibyo Gakkai Zasshi.

[13] Tey YQ, Ravi K, Chong CS, Chiong E, Ho J, Tey JC, Ho F (2020). Management of locally advanced synchronous colorectal and prostate cancers: A case report. Medicine (Baltimore).

